# SARS-CoV-2 Among Migrants Recently Arrived in Europe From Low- and Middle-Income Countries: Containment Strategies and Special Features of Management in Reception Centers

**DOI:** 10.3389/fpubh.2021.735601

**Published:** 2021-11-30

**Authors:** Silvia Fabris, Gabriella d'Ettorre, Ornella Spagnolello, Alessandro Russo, Maurizio Lopalco, Fausto D'Agostino, Paolo Vassalini, Luigi Celani, Raissa Aronica, Simona Gabrielli, Gabriele d'Ettorre, Silvia Angeletti, Claudio M. Mastroianni, Massimo Ciccozzi, Giancarlo Ceccarelli

**Affiliations:** ^1^National Center for Control and Emergency Against Animal Diseases and Central Crisis Unit - Office III, Directorate General for Animal Health and Veterinary Drugs, Italian Ministry of Health, Rome, Italy; ^2^Medical Statistics and Epidemiology Unit, Campus Bio-Medico University, Rome, Italy; ^3^Department of Public Health and Infectious Diseases, Sapienza University of Rome, Rome, Italy; ^4^Infectious Diseases Unit, Azienda Ospedaliero-Universitaria Policlinico Umberto I, Rome, Italy; ^5^Department of Medical and Surgical Sciences, “Magna Græcia” University, Catanzaro, Italy; ^6^Medical Center, Extraordinary Reception Center “Mondo Migliore”, Rocca di Papa, Italy; ^7^Italian Ministry of Health, Rome, Italy; ^8^Anesthesia and Resuscitation Unit, Campus Bio-Medico University, Rome, Italy; ^9^Infectious Diseases Unit, Belcolle Hospital, Viterbo, Italy; ^10^Unit of Parasitology, Azienda Ospedaliero-Universitaria Policlinico Umberto I, Rome, Italy; ^11^Unit of Occupational Prevention and Protection, Azienda Sanitaria Locale Lecce, Lecce, Italy; ^12^Unit of Clinical Laboratory Science, University Campus Bio-Medico of Rome, Rome, Italy; ^13^Migrants and Global Health Research Organization, Rome, Italy

**Keywords:** COVID-19, SARS-CoV-2, outbreak, migrant reception center, asylum seekers and migrant, global health, infection prevention and control, surveillance

## Abstract

Despite the “migrants and COVID-19” topic has been neglected since felt marginal concerning other aspects of the SARS-CoV-2 pandemic, it represents a relevant public health issue in the European countries. This report describes COVID-19 containment strategies adopted in a large Italian reception center hosting recently arrived asylum-seeker migrants. Risk assessment and prevention measures adopted were described. Geo-spatial epidemiological analysis of the outbreak was reported. Significant gaps in the knowledge of self-protection measures from contagious diseases and in the perception of the pandemic risk were observed in migrants; health promotion activities, targeted to remove cultural barriers and improve behaviors appropriate to individual protection, were able to fulfill this gap. In low-resource settings, especially in closed communities, the implementation of social distancing strategies, the systematic use of individual protection devices, and active syndromic surveillance are essential tools to limit the risk of outbreaks. In the event of an outbreak, it is relevant to rapidly activate containment procedures based on systematic screening, isolation, and quarantine, taking into consideration the limits of tracing contacts within a closed community. Not being able to trace certain contacts, the geo-spatial epidemiological analysis of cases distribution could be key in the management of the outbreak. Interestingly, positive cases identified in our facility were all clinically pauci-symptomatic or asymptomatic. Dedicated strategies are needed to minimize the chance of SARS-CoV-2 transmission in a limited space such as reception centers and a vulnerable population such as migrants.

## Introduction

Overall, 2.7 million immigrants entered the European Union (EU) from non-EU countries in 2019, mainly low- and middle-income countries. Italy, traditionally characterized by considerable migratory flows, has been one of the hardest-hit European countries by the severe acute respiratory syndrome coronavirus 2 (SARS-CoV-2) pandemic (1–3). Migrants living in refugee camps, detention centers, and reception centers are particularly vulnerable to SARS-CoV-2 infection (4). Individuals living in such overcrowded setting are indeed less keen on following the basic prevention practices including social distance, hand hygiene, and self-isolation in case of exposure to SARS-CoV-2.

The management of coronavirus disease 2019 (COVID-19) risk for newly arrived migrants hosted in reception centers presents many challenges, mainly linked with the cultural and linguistic heterogeneity of this specific population. Previously, heterogeneity in the perception of the risk related to COVID-19 and in the compliance with preventive measures to reduce the risk of transmission was reported among migrants coming from different countries (5, 6).

Despite this being a relevant public health topic, currently, a small number of reports have focused on the problem and, to the best of our knowledge, no outbreak has been described in detail in reception centers for migrants, creating consistent knowledge and data gap.

In this report, we described a SARS-CoV-2 outbreak in a large Italian reception center hosting recently arrived asylum seeker migrants and analyzed the prevention methods and containment resources used to manage the epidemic.

## Methods

### Setting

**Figure 1:**
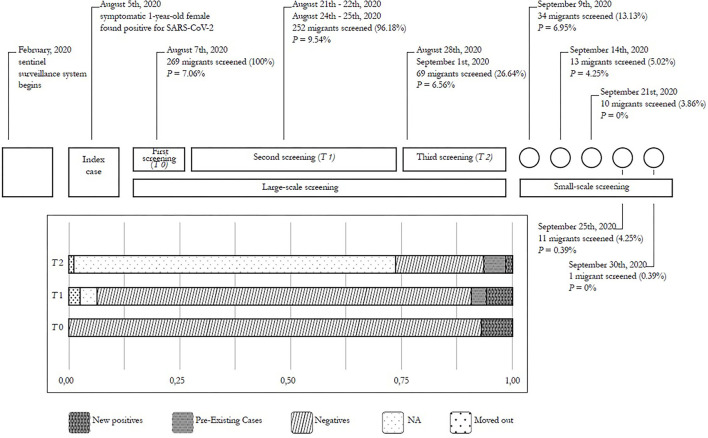
Timeline of the ERC COVID-19 outbreak.

From February to September 2020, the Extraordinary Reception Center (ERC) “Mondo Migliore” of Rocca di Papa (Rome) overall hosted 355 asylum seekers largely from North Africa, the Gulf of Guinea, the Horn of Africa, Syria, Pakistan, and Bangladesh. In the period August–September 2020, 305 migrants were housed. The center was located on the outskirts of Rome. People hosted had no restriction for entry and exit, while non-resident people or those who did not work in the structure were not allowed. The facility could therefore be considered a pool of migrants that interacts with the local population only outside the center and that remains completely isolated in case of quarantine. The ERC is located in a large building (over 500 beds capacity) with modular architecture consisting of separate housing sectors joined together through shared spaces (corridors and lounges). Moreover, the center is equipped with single, double, and triple rooms, a canteen and a common room for having meals, a television room, a shared space for meetings, a mosque, and a Christian church. A park for physical activity surrounds the center (map is shown in Figure 1). A legal office, a social service, and a clinic were available for free. The medical clinic, managed by a team of physicians accredited in internal medicine and infectious diseases and nurses, was open 365 on 365 days, 6 h/daily. The staff members (formally called ERC workers) were a total of 36 people.

### Clinical Evaluation and Data Collection

Medical screening was performed on arrival for all migrants to exclude pathologies that prevent living in the community and to assess health needs of the guests (7–11). Clinical history of all migrants was obtained by an individual interview with the support of a cultural mediator. Check-ups were delivered on a voluntary basis to all guests, while follow-up assessments were dedicated to patients with acute or chronic conditions. Clinical data, blood analysis, and radiological tests were recorded in clinical files and stored on an electronic database.

### Sentinel Surveillance System for Early Outbreak Detection and Case Definition

An epidemiological surveillance service for communicable diseases was up and running in the center and based on the collaboration between the infectious disease specialist, an epidemiologist, and the territorial service of the local government health company (12–16).

About the management during the SARS-CoV-2 pandemic, all newly arrived hosts were allowed if asymptomatic and testing negative at an antigenic or molecular nasopharyngeal swab (NPS) was performed in the prior 48 h. None of the newly arrived migrants was subjected to anti-SARS-CoV-2 antibody assessment. The management of migrants before entering the reception centers provides for 10 days of quarantine, active surveillance for monitoring the health status of the guests, with body temperature detection two times a day, execution of molecular tests in case of the onset of symptoms, and execution of two antigen tests, one at the entrance and the second at the end of the quarantine.

Since monitoring with molecular and/or antigenic NPS and serological tests was not feasible, the strategy adopted by the medical staff was: (1) to implement health promotion, training guests on prophylaxis procedures, (2) regular distribution of facial masks and handwashing with hydroalcoholic gel dispensers, (3) regular check of body temperature and clinical monitoring with the referral to the Emergency Department (ED) of all patients with symptoms keeping with COVID-19 or influenza-like illness (ILI) (17, 18), (4) hosting guests in single rooms, whereas multiple rooms were used only for families, and (5) social distancing in the shared areas of the center (5).

To evaluate the attitude of migrants to observe SARS-CoV-2 preventive measures inside the center, medical staff surveyed by semi-structured interviews aiming to investigate people self-perception of the risk related to the pandemic and to assess the knowledge on the prevention tools. The “Standard questionnaire on risk perception of an infectious disease outbreak” was tailored to address COVID-19 and was translated into the different predominant languages spoken in the center (19). This tool was designed to study risk perceptions associated with the outbreak of infectious disease and has been used in many previous studies (20, 21).

A confirmed case of SARS-CoV-2 infection was based on the grounds of a positive Nucleic Acid Amplification Test for SARS-CoV-2, following the WHO COVID-19 Case definition.

### Statistical Analysis

We carried out an explanatory and descriptive analysis about migrant guests and demographic features of ERC workers. Then, we compared immigrant characteristics among those who tested positive at least once during the outbreak and those who were never found positive, by Wilcoxon's test and Chi-square test; we tested if the proportion on positives was significantly smaller than negatives by *Z*-test. We displayed the evolution of the outbreak by large-scale and small-scale screening. In particular, we studied attack rates (*AR*) at *T0* to identify potential risks. We assessed significant associations between potential factors and testing positive for SARS-CoV-2 by Chi-Square test and, once we found an association, we computed the risk ratios (*RR*) to quantify risks. We defined the neighborhood structure of ERC areas to study the spatial autocorrelation of *AR* at *T0* by Global Moran I. We investigated if the measures taken were effective in containing the epidemic, by calculating Incidence Ratio (*IR*) and Incidence Rate Ratio (*IRR*) at *T1*. Through logistic regression, we observed how the significant risk factors identified affected the population before and after guests were isolated and quarantined. Tests and parameter significance were evaluated with α = 0.05.

### Ethics

This study is a description of the events, and of the epidemiological and clinical practice adopted to control the pandemic. Data were obtained during the epidemiological investigation by the Latium Regional Health Authority and Local Health Authority, to identify/contain an ongoing epidemic cluster, to provide recommendations, to prevent new outbreaks, and to avoid complications in the infected subjects. The approval of the Institutional Review Board and Ethical Committee was not required since it is not necessary based on the current Italian legislation; moreover, we operated under emergency circumstances. The study was performed in accordance with good clinical practice and the declaration of Helsinki.

## Results

### COVID-19 Surveillance and Prevention Measures

Following the SARS-CoV-2 outbreak in China and the first case of COVID-19 recorded in Italy, from March 9, 2020 to May 18, 2020, the Italian government imposed a national quarantine, severely restricting movements of the population except for necessity, public service work, and health circumstances, in response to the spread of the virus in the country. In this period, a total lockdown was in place in the ERC and only staff members were allowed to enter and exit under strict clinical monitoring and with the mandatory use of personal protective equipment. Subsequently, based on government provisions and as a consequence of the decrease in the number of COVID-19 cases on the national territory, the quarantine was interrupted. In this period, from the end of February to July 31, 2020, no cases of symptomatic SARS-CoV-2 infection were recorded in the ERC. Since the systematic monitoring with molecular and/or antigenic NPS and serological tests was not available within the center for the people housed, we are unable to prove that there were no asymptomatic cases undiagnosed. However, we observed that all the 102 (102/355; 28.7%) migrants “randomly” tested by antigenic or molecular NFS from February to July 31, 2020, were negative. Most of these tests were performed (1) voluntarily as personal screening or (2) upon the request of local clinics and hospitals to access the facilities. All the ERC workers were repeatedly negative in molecular screening tests required to get access to the center.

Moreover, social distancing was imposed in the common areas of ERC and the canteen, with the obligation to adopt face masks and facilitating access to hydroalcoholic gel dispensers.

Before asking migrant guests to observe SARS-CoV-2 preventive measures inside the center, medical staff carried out a survey aiming to investigate the self-perception of people on the risk related to the pandemic. The most relevant and critical issue was to assess the awareness of the prevention tools by semi-structured interviews. The results showed that the risks associated with pandemic were poorly perceived among guests, mainly among those from Guinea gulf area. Moreover, a significant gap in the knowledge of the basic procedures indicated to protect themselves from a respiratory/contact transmissible disease was observed in migrants coming from sub-Saharan Africa. Consequently, a health-promotion intervention was carried out to provide information on adequate behaviors to avoid contagion: small groups of guests, homogeneous for cultural context, were invited to take part in multiple meetings in which the risks of the pandemic were explained and virtuous prevention behaviors encouraged. With the help of cultural mediators, the message was repeatedly reinforced over time.

### Description of Outbreak, Management, and Control Measures

In August 2020, 305 persons were living in ERC; out of them, 269 were immigrants coming from 28 different countries (mainly from Africa). Of them, 70.6% were male and the median age was 26 years (the youngest was younger than 1 year and the oldest was 67 years of age); more specifically the 17.84% of migrants were minors (with a median age of 4 years). Thirty-six were ERC staff members, 83.3% coming from Italy and 16.7% from other countries. The median age of ERC workers was 35.5 years, ranging from a minimum of 23 to a maximum of 51 years.

On August 5, 2020, a 1-year-old girl from Nigeria (index case), taken to the hospital because of fever, was found to be positive for SARS-CoV-2 at NPS; consequently, all of the reception center workers and residents were quarantined. On August 7, all of them underwent baseline SARS-CoV-2 screening (*T0*): one staff member and 18 guests resulted positive, leading to an initial *AR* of 2.778% and 7.063% in the two populations, respectively. During this time span, the risk of getting infected among immigrants was 2.54 times higher than among ERC workers. However, 95% CI (0.35–18.43) revealed that the risk was not significantly higher among immigrants (*p* = 0.33). Once individuals were found positive, they were moved to an isolated sector of the center (area S) to be separated from negative residents and complete the quarantine. Interestingly, all subjects tested positive had a vague respiratory symptomatology (47.3% flu-like) or were asymptomatic (52.7%).

Between 14 and 18 days later, 252 migrants in the reception center were tested for SARS-CoV-2 for the second time (*T1*). Among guests positive on *T0*, 9 kept testing positive for SARS-CoV-2. Moreover, the *T1* screening revealed 16 new asymptomatic cases.

On August 28 and on September 1, only part of the population was tested again (*T2*): 190 were not screened and 3 were transferred to other reception centers. Four new asymptomatic cases were detected (3 on the 28th and 1 on the 1st), for a total of 20 new positive cases from *T0*. Results of this screening process are summarized in Figure 1.

A smaller number of immigrants were tested several times (on September 9, 14, 21, 25, and 30): new cases were not reported during this month. On average, guests who stayed at the reception center were found negative after 20.7 (± 13) days from when they had been declared positives, ranging from a minimum of 3 to a maximum of 54 days. Positive guests resulted positive on average 2.72 ± 1.97 times (from a minimum of 1 to a maximum of 8) before turning negative.

### Epidemiological and Clinical Characteristics of Infected Patients

The rate of guests testing positive at least once was significantly smaller than the one who never caught SARS-CoV-2 (*p* < 0.001). Focusing on migrants testing positive for SARS-CoV-2, they were 23-year-old in median (ranging from 0 to 61 years), 7 minors (17.5% of them) were found positive during the outbreak. Guests came from 13 different countries (30% from Nigeria, 12.5% from Bangladesh, 10% from Ghana, and 47.5% from the remaining countries) and most of them (62.5%) were men. However, none of these characteristics resulted significantly associated with a positive test or were different from the ones observed among negative guests with a confidence level of 95%, except for the nationality. Results are summarized in Table 1.

**Table 1:** Descriptive statistics: all guests, positive guests, and negative guests; tests and related *p*-value.

	**Total**	**SARS-CoV-2**		**Test**	***p*-value**
		**Positive**	**Negative**		
N° subjects	269	40	229	*Z*-test	<0.001
Mean age (years)	25.8 (13.1)	23.4 (13.6)	26.2 (13)	Wilcoxon	0.1451
N° males (%)	190 (70.6)	25 (62.5)	165 (71.1)	Chi-square	0.3003
N° subjects <18 years (%)	48 (17.8)	7 (17.5)	41 (17.9)	Chi-square	0.3402
N° males <18 years (%)	26 (54.2)	2 (28.6)	24 (58.5)	Chi-square	0.561
Nationality	–	–	–	Chi-square	0.04367

From a clinical point of view, the majority of SARS-CoV-2 positive patients remained asymptomatic except for the “index case,” which presented fever in the absence of other complications and 9 other subjects screened in the first wave who presented with a modest flu-like syndrome (characterized mainly by rhinorrhea and headache). No patient required hospitalization for worsening clinical conditions and all had a completely benign disease course.

### Spatial Distribution of Clusters and Risk of SARS-CoV-2 Infection

**Figure 2:**
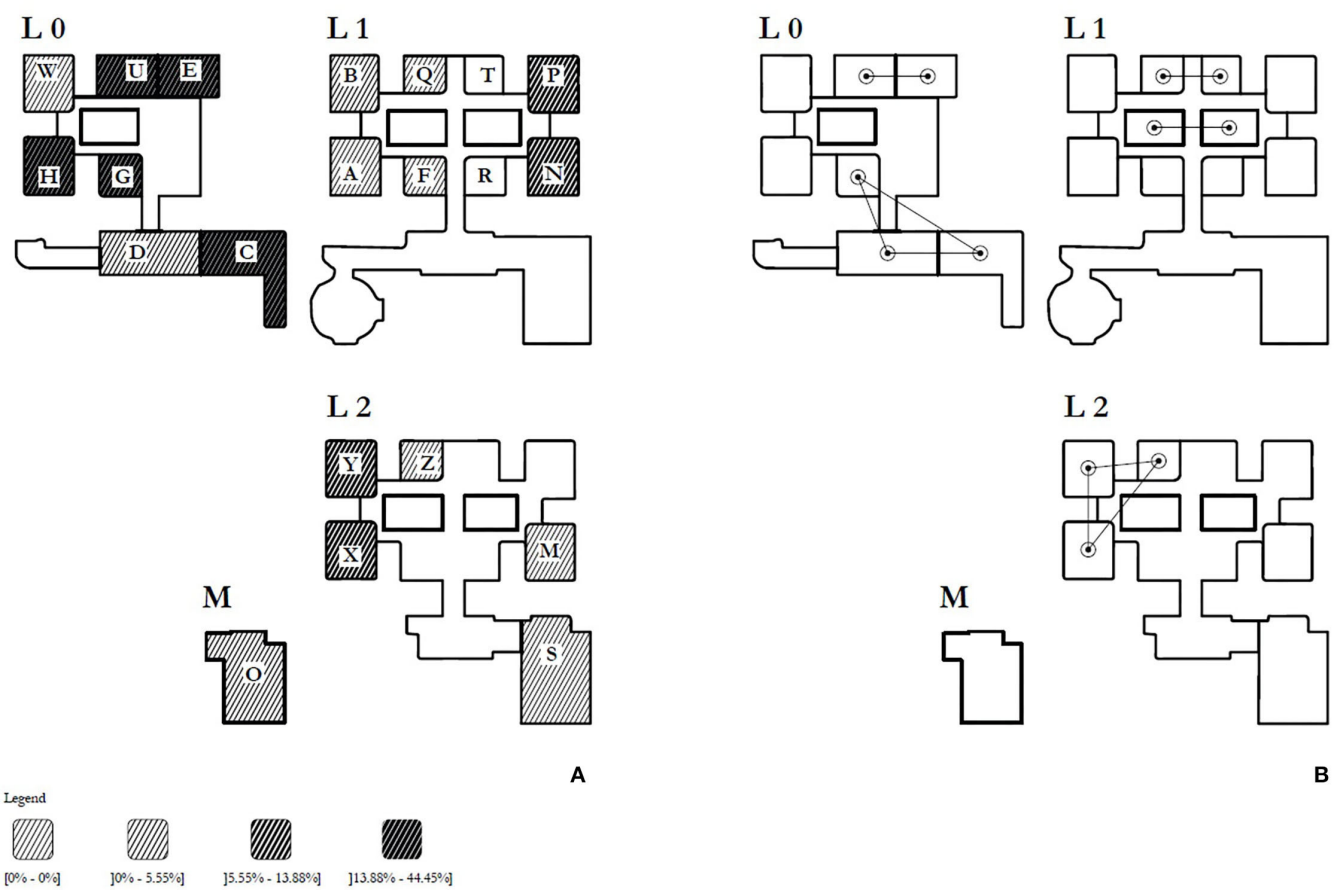
Map of the building hosting the migrants. **(A)** Spatial distribution (by quartiles) of the attack rates per hundred over the time period between the identification of the first case and tests. **(B)** neighborhood structure of ERC.

Out of 269 immigrants, information about levels and areas of the center where guests were located are certainly available for 264. Using these records, we were able to compare *AR*s in different areas of the ERC: risks of testing positive for SARS-CoV-2 on *T0* was calculated for each area separately and then plotted, by quartiles, in Figure 2A.

On one hand, Figure 2A shows higher risks in certain areas located at Level 0. In particular, the most affected areas are C, where the 1-year-old girl stayed with her family (*AR* = 44.4%), E and G (*AR* = 20%), U and H (*AR* = 16.7%); on the same Level, area W is the only one that was coronavirus-free. We investigated the association between testing positive for SARS-CoV-2 at *T0* and living in Level 0 and then, more specifically, in area C: among the 75 persons assigned to Level 0, 11 were positive for SARS-CoV-2 at *T0* (*AR* = 14.67%), showing a significant association between Level 0 and positivity (*p* = 0.007): more specifically the risk of being infected for who lived at Level 0 (*RR*) is 3.465 times higher than among who lived on others Levels (95% CI: 1.451;8.275, *p* = 0.003). Being assigned to area C was significantly correlated with testing positive for SARS-CoV-2 at *T0* both with respect to all other areas and areas located at Level 0 (*p* < 0.001 and 0.02, respectively). In fact, the risk among people living in C (*RR*) was 7.556 times (95% CI: 3.133; 18.217, *p* < 0.001) and 4.190 times (95% CI: 1.523; 11.528, *p* = 0.007) higher compared with the risk of those living in other areas and Levels and other Level 0 areas, respectively. On the other hand, Figure 2B shows the neighborhood structure: it was defined as bordering areas of the building sharing access roads and spaces for social aggregation. There are 19 inhabited areas, 11 of them are isolated and 8 are connected with a maximum of two neighbors. The Moran I highlights a low spatial autocorrelation equal to 0.187.

### Social Behavior and Risk of SARS-CoV-2 Infection

To investigate if certain social behaviors could enhance the spread of SARS-CoV-2 and raise the risk of being infected, we identified a group of closely related Nigerians, composed of 11 women and their 5 sons, including the 1-year-old girl who was *SARS-CoV-2* positive (index case) and her mother who was not found positive on August 7. Seven of them, plus the index case, were positive to SARS-CoV-2 highlighting that there is a correlation between being part of a close group and testing positive to the virus (*p* < 0.001). Moreover, the risk of being infected in this group (*RR*) is 12.23 (95% CI: 6.729–22.219) times significantly higher than among the other guests.

**Figure 3:**
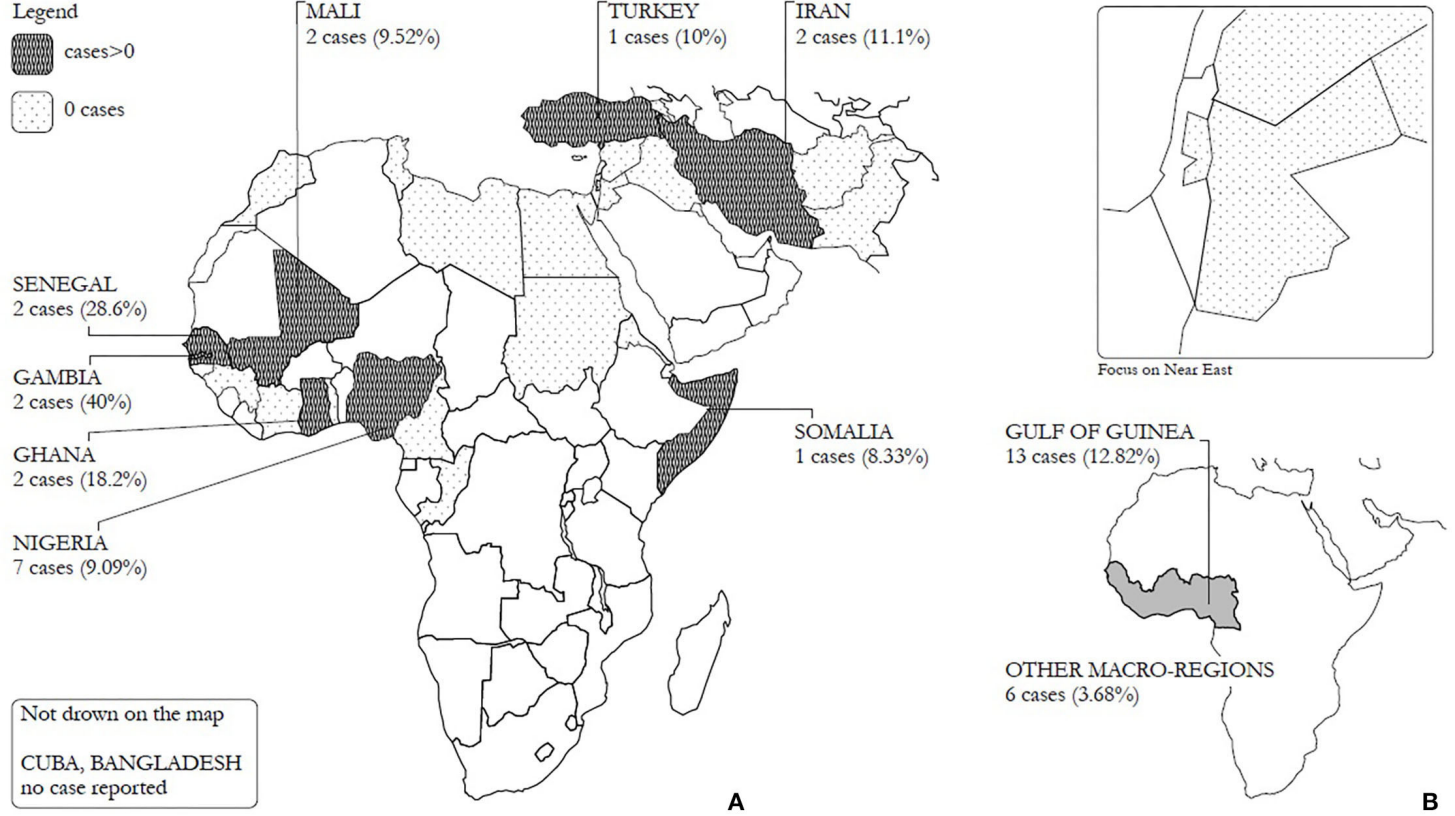
**(A)** Number of cases (*AR*%) registered by nationality and **(B)** number of cases (*AR*%) registered by macroareas.

The 36.85% of immigrants found positive at *T0* came from Nigeria (including the index case), the remaining 63.15% came from seven different countries (Gambia ≃10.5%, Ghana ≃10.5%, Iran ≃10.5%, Mali ≃10.5%, Senegal ≃10.5%, Somalia ≃5.26%, and Turkey ≃5.26%) We computed the *AR* separately for each nationality where the immigrants come from, in order to observe risks (Figure 3A). On a macro-regional level, Nigeria, Gambia, Ghana, and Senegal are part of the Gulf of Guinea (Figure 3B): there was a significant correlation between testing positive at *T0* and belonging to this area, compared to the other ones (*p* = 0.015). More specifically immigrants from this macro-region had a risk (*RR*) 3.332 times higher than other guests (95% CI: 1.307; 8.494, *p* = 0.007).

### Effect of the Measures Taken in Containing the Epidemic

Among people tested at T1, 12 of them were located in those areas where there were positives at T0 (IR = 3.97 per 1,000 persons—day) the remaining 3 came from SARS-CoV-2-free areas (IR = 2.77 per 1,000 persons—day). The IRR shows that the risk among those who lived in the same area of an infected person was not statistically higher than the one among those living in a SARS-CoV-2-free area (IRR = 1.434, 95% CI: 0.405–5.081). The most significant risk factors to the spread of the virus are (1) living in area C, where the outbreak has been detected for the first time and (2) the macro-regions where guests come from. According to those factors, we divided the hosts on the basis of (1) the area of residence (resident in area C of the building or not), and (2) the macro-region of provenience (born in the Gulf of Guinea region or not). We explained how this risk affected being tested positive on August 7, when the population was free to move in the ERC, and lately, when positive guests were isolated in area S and negatives were quarantined in their rooms: logistic regressions details are reported.

logit (being tested positive on August 7) = −3.3914 + 1.1705(coming from Gulf of Guinea) + 2.3615 (being assigned to C); *p* < 0.001, = 0.0258, = 0.0015, respectively.

The same arguments do not explain the probability of being tested positive after the implementation of containment measures: coming from Gulf of Guinea *p* = 0.639 and being assigned to area C *p* = 0.959.

## Discussion

To the best of our knowledge, this is the first comprehensive report available on this topic, even though it has now been over a year since the beginning of the SARS-CoV2 pandemic. In fact, although some data are generally available on migrants, nothing has been reported on asylum seekers housed in reception centers. This underlines how the “migrants and COVID-19” topic is still neglected and marginal with respect to the management of other aspects of the pandemic that are currently considered more urgent and relevant (22, 23). Despite this, the topic remains extremely challenging and represents a relevant public health issue taking into consideration that guests of migrant reception centers live in a relatively close community, where individuals are meant to share common spaces and time (i.e., recreational, religious, and cultural activities) but are still free to leave the ERC and mingle with the local population.

The most intriguing aspects of the events described were represented by (1) the long period (from February to July 2021) free from SARS-CoV-2 cases within the ERC, (2) by the rapid containment of the outbreak despite it took place in a closed community vulnerable to a rapid spread of the virus, and (3) the absence of clinical symptoms of almost all infected migrants.

Surveillance and prevention measures aiming to limit the risk of a SARS-CoV2 outbreak taken in place in the ERC contributed to minimize the risk of onset of COVID-19 cases within the center from February to July 2020 (5). In fact, the center remained free from cases of SARS-CoV-2 infection both during the national lockdown and for more than 2 months afterward, in contrast to what was simultaneously observed in many other closed communities (such as in religious centers, schools, hospitals, and barracks). Taken together, this evidence also suggests that migrant's adherence to preventive measures adopted in the ERC were rock-solid. In particular, since the beginning of the pandemic, as previously reported, the medical team worked on the following key aspects: (1) to assess the awareness on the risks related to the epidemic and on the prevention tools by semi-structured interviews, (2) to promote consequent health interventions carried out to provide information on correct behaviors to avoid contagion, (3) to adopt a strategy based on full-time face mask use, hydroalcoholic gel dispenser wide availability plus a “pushed” social distancing (with single rooms in most cases), and (4) to actively monitor the appearance of any symptoms possibly related to COVID-19 (5, 24).

The knowledge gap relating to prophylaxis procedures and the low perception of the risk initially observed in our ERC population agree with what was reported by a recent document of the International Organization for Migration. In fact, this report underlined that the particular vulnerability of migrants toward COVID-19 is linked to (1) the limited awareness of recommended prevention measures, including due to linguistic barriers, (2) the inability to respect social distancing in crowded, multigenerational homes, and (3) the limited access to key hygiene items (25).

Interestingly in our case, although a large majority of migrants (especially among sub-Saharan people) initially presented significant gaps in the knowledge of the basic prophylactic procedures for respiratory transmissible disease, the health promotion interventions carried out with the help of cultural mediators led to an acceptable compliance to individual and collective protection procedures (5).

In August 2021, the outbreak was discovered thanks to the attention paid to a modest symptomatology compatible with ILI in an infant, which was investigated by molecular NPS for SARS-CoV-2. The immediate response with the quarantine for the whole ERC and the execution of NPS on all guests was adopted. This strategy allowed the medical team to identify the positive cases and to separate them from the remaining hosted population by isolating them in a dedicated area. Despite the quarantine being already ongoing, this decision was taken to strictly monitor the respect for isolation precautions: in fact, the belief that the absence of clinical symptoms meant absence of disease (and of risk of contagion) was widespread in some ethnic groups, mainly in Nigerians. Based on those measures, the number of new positive cases progressively decreased in the subsequent screenings carried out to identify the infected subjects among the contacts of the already known positive migrants. Also, in this case, all ERC hosts were tested with molecular NPS, not being able to trace certain contacts within a closed community.

Prompt SARS-CoV-2 screening by NPS of the whole population at baseline was crucial to identify clusters of individuals testing positive in order to investigate local risk factors to be carefully monitored. Of these, allocation to a specific area of the RC and belonging to a particular ethnic group.

Interestingly, the allocation of guests in separate sectors of the building limited the spread of the outbreak, despite the use of common living areas (prayer rooms, canteen, television room, and shared space for meetings). Probably this result can be explained considering that the family setting is the one in which it is more likely to lower the attention on preventive measures, whereas in the community, social distancing and the use of face masks is more common. Characteristically, in the building sectors, a micro-community can frequently arise within which material and human resources are shared (inter-family help and friendships). Although guests are accommodated in single rooms, social contacts can take place in this “familiarly” context with little attention to individual protection procedures. These results underline that geo-spatial analysis of the distribution of cases is a key resource in the management of the outbreak. Similarly, Gorny et al. (26) reported that the number of cases of SARS-CoV-2 infection in migrant worker dormitories in Singapore doubled every 1.56 days in barracks-style buildings and in 2.65 days in apartment-style buildings, suggesting that building design plays a crucial role in the spread of contagious diseases. Therefore, the plan of a building and its structure are not the only determinants of the risk of spreading an outbreak within a closed migrant community. In the early stages of the spread of the disease, when restrictive or containment measures are not yet in place, social relations between people and the commonality of culture, religion, and language (which often represent factors of aggregation beyond the spatial location of the person in the building) are further key factors.

Finally, among all COVID-19 cases recorded at the ERC, only the “index case” and a minority of patients had mild upper tract respiratory symptoms: the remaining cases were fully asymptomatic. This clinical feature can be linked to specific factors (such as possible low viral load, hot temperatures in the summer season that reduces the impact of respiratory diseases, intrinsic host resistance) or recognize a multifactorial origin.

Several studies focused on the impact of ethnicity on SARS-CoV2 susceptibility; however, on the basis of the currently available data, it is unclear how genetic characteristics may modulate susceptibility to SARS-CoV-2 and the severity of the infection. Among acquired factors to be evaluated, disproportionate socio-economic and environmental stressors have been proposed as possible determinants of poor attitude to practice physical distancing in Black, Indigenous, and People of Color communities and causes of decreased viral immune defense and increased susceptibility to SARS-CoV-2 infection with substantial risk of severe illness (27–29). On the other hand, a diet able to maintain a balanced and rich in lactobacilli microbiome has been proposed as a protective agent against COVID-19 progression (30–35). This suggestive hypothesis remains unproven, but we noticed that the daily intake of milk products was high in our specific population, suggesting further microbiome comparative studies to prospectively evaluate if this factor might play a role in the observed mild course of the disease.

Major limitations of this report were the unavailability of (1) a pre-outbreak screening program with molecular o antigenic NPS, (2) a pre-outbreak screening with serological tests to evaluate the percentage of population prone to infection, (3) a post-outbreak screening with serological tests to evaluate antibody responses to SARS-CoV-2 infection. Moreover, we evaluated the effectiveness of health promotion interventions dedicated to the implementation of prophylactic measures by observing a significant increase in the use of face masks and hydroalcoholic gels, but no further questionnaires or interviews were carried out to measure the level of adherence to preventive measures.

On the other hand, this report drew up in a limited resource intervention setting, represents an almost unique model of management within a semi-closed migrant community, and focuses on still poorly explored fragile populations.

## Conclusions

Our report raises up the awareness of how migrant reception centers have been affected by the global COVID-19 pandemic, going through many challenges mainly related to different cultural backgrounds of the given population, and stresses the relevance of (1) a systematic screening addressing the whole ERC population, (2) tracking of the index cases, and (3) prompt preventive measures in place.

Interestingly, despite all our guests having significant gaps in basic knowledge of how to protect themselves from respiratory transmissible disease, sub-Saharan migrants were particularly unaware of the given topic. These data stress the cultural background and heterogeneity of an ERC population, which should be thoroughly considered to predict the compliance of preventive measures to be taken.

Moreover, our report outlined how cultural behaviors have also an impact in terms of SARS-CoV-2 transmission. Some ethnic groups, indeed, are more prone than others in clustering and this represents a considerable risk factor for infection, similar to the allocation in a certain area of the ERC. As evidenced by this and previous reports, geo-spatial epidemiology is becoming increasingly important in the control of outbreaks in closed communities: migrant reception center management should always consider adequate host allocation strategies designed to reduce the risk of spreading contagious diseases. Staff members should bear in mind all these concepts in planning a proper response to minimize the chance of SARS-CoV-2 transmission in such a confined and epidemiological intricated space as ERCs.

## Data Availability Statement

The raw data supporting the conclusions of this article will be made available by the authors, without undue reservation.

## Ethics Statement

Ethical review and approval was not required for the study on human participants in accordance with the local legislation and institutional requirements. The patients/participants provided their written informed consent to participate in this study.

## Author Contributions

SF and GC: conceptualization. SF, GC, OS, Gabriellad'E, and Gabrieled'E: methodology. GC, FD'A, SG, RA, PV, LC, ML, and SA: resources and data curation. SF, GC, and OS: writing—original draft preparation. GC, MC, CM, AR, and Gabriellad'E: writing—review and editing. GC, MC, Gabriellad'E, and CM: supervision. All authors have read and agreed to the published version of the manuscript.

## Conflict of Interest

The authors declare that the research was conducted in the absence of any commercial or financial relationships that could be construed as a potential conflict of interest.

## Publisher's Note

All claims expressed in this article are solely those of the authors and do not necessarily represent those of their affiliated organizations, or those of the publisher, the editors and the reviewers. Any product that may be evaluated in this article, or claim that may be made by its manufacturer, is not guaranteed or endorsed by the publisher.
